# Supplementary material for: Cannabidiol Effectively Promoted Cell Death in Bladder Cancer and the Improved Intravesical Adhesion Drugs Delivery Strategy Could Be Better Used for Treatment
Source: Pharmaceutics. 2021 Sep 7;13(9):1415. doi: 10.3390/pharmaceutics13091415 (PMC8471856; doi:10.3390/pharmaceutics13091415)
Supplement: Supplementary file 1 [file pharmaceutics-13-01415-s001.zip › pharmaceutics-1332169-supplementary.pdf]

pharmaceutics

Supplementary Materials: Cannabidiol Effectively Promoted Cell Death in Bladder Cancer and the Improved Intravesical Adhesion Drugs Delivery Strategy Could Be Better Used for Treatment

Shanshan Chen, Changping Deng, Wenyun Zheng, Shihui Li, Yuping Liu, Tong Zhang, Chen Zhang, Yunhui Fu, Hui Miao, Fuzheng Ren and Xingyuan Ma

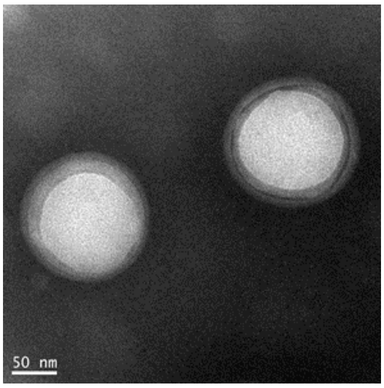

Figure S1. TEM images of blank PLGA NPs.

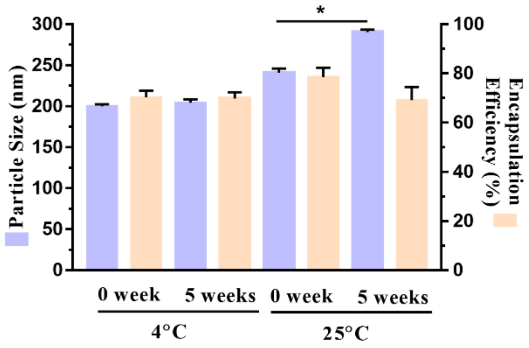

Figure S2. Changes in particle size and EE of CBD-loaded PLGA NPs at 4 °C and 25 °C for 2 weeks and 5 weeks.

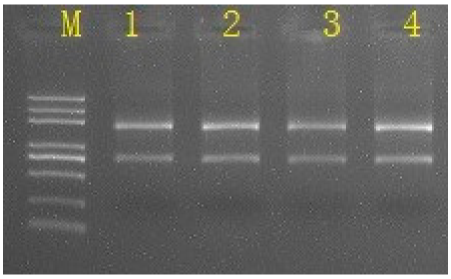

Figure S3. RNA extraction from CBD-treated T24 cells for transcriptome sequencing.

Table S1. The biological characteristics of Human T24, 5637, UM-UC-3, and SV-HUC-1 cell lines.

Cell	T24	UM-UC-3	5637	SV-HUC-1
Organism	*Homo sapiens*			
Morphology	epithelial			
Tissue	Urinary bladder			Ureter; Uroepithelium
Growth properties	Adherent			
Gender	Female	Male		
Disease	Transitional Cell Carcinoma		Grade II Carcinoma	No
Tumorigenic	Yes, in hamster cheek pouch; No, in nude mice	Yes, Tumors developed within 21 days at 100% frequency (5/5) in nude mice inoculated subcutaneously with 10^7 cells.	Yes, within 21 days at 100% frequency (5/5) in nude mice inoculated subcutaneously with 10^7 cells.	No, nude mice

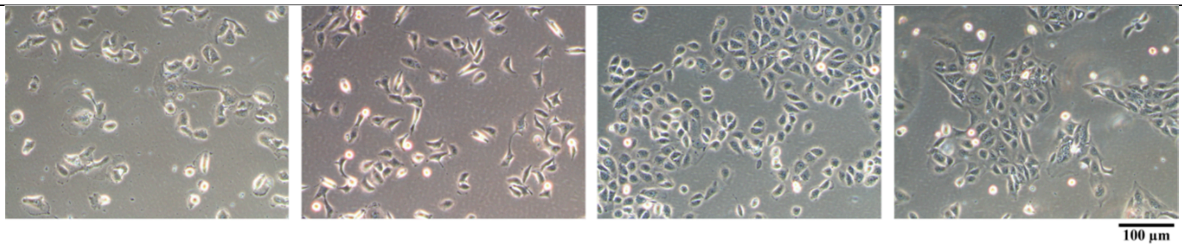

T24 **UMUC-3** **5637** **SV-HUC-1**

Table S2. Global transcriptome database sequencing data statistics table.

Sample	Raw reads	Clean reads	Error rate (%)	Q20 (%)	Q30 (%)	GC content (%)
1	44539156	44342584	0.0274	96.95	92.02	51.60
2	52773994	52458958	0.0275	96.93	91.98	50.78
3	48162960	47939354	0.0277	96.82	91.78	51.10
4	55023994	54737156	0.0282	96.62	91.37	49.91

Table S3. The primers used for qPCR analysis.

Primer	Forward sequence (5′-3′)	Reverse sequence (5′-3′)
β-actin	ATTGGCAATGAGCGGTTC	GGATGCCACAGGACTCCAT
Caspase 7	GAATGGGTGTCCGCAACG	TTGGCACAAGAGCAGTCGTT
Bax	TGCGTCCACCAAGAAGC	TCCAGTTCGTCCCCGAT
Bcl-2	GCGGATTGACATTTCTGTG	CATAAGGCAACGATCCCA
Cyto-c	GCTAAACACCAGGACGGAACT	CCACTCCCAATCAGGCATGAAC
MMP2	ACCTGGATGCCGTCGTGGAC	TGTGGCAGCACCAGGGCAGC
MMP9	CACTGTCCACCCCTCAGAGC	GCCACTTGTCGGCGATAAGG

Table S4. The characterization of NPs.

Nanoparticles	CBD/PLGA NPs	CS-CBD/PLGA NPs
Size (nm)	192.9 ± 2.41 nm	287.2 ± 0.9 nm
Zeta potential (mV)	-6.27 ± 0.927 mV	3.37 ± 0.158 mV
PDI	0.041 ± 0.027	0.1335 ± 0.025
